# Rapid changes in mucociliary transport in the tracheal epithelium caused by unconditioned room air or nebulized hypertonic saline and mannitol are not determined by frequency of beating cilia

**DOI:** 10.1186/s40635-021-00374-y

**Published:** 2021-03-17

**Authors:** Susyn Joan Kelly, Paul Martinsen, Stanislav Tatkov

**Affiliations:** 1 Fisher & Paykel Healthcare Limited, 15 Maurice Paykel Place, East Tamaki, 2013 Auckland, New Zealand; 2 Blue Leaf Software Limited, Hamilton, New Zealand

**Keywords:** Mucociliary transport, Cilia, Mucus, Video-microscopy, Airway epithelium, Humidity, Nebulizer, Hypertonic saline, Mannitol

## Abstract

**Background:**

Inspired air is heated and humidified in the nose before it reaches lower airways. This mechanism is bypassed during tracheostomy, directly exposing the airways to colder and drier air from the environment, known to negatively affect mucociliary transport; however, little is known about how quickly mucociliary transport deteriorates. This study determines the short-term effect of flowing room air and nebulized hypertonic saline and mannitol on mucociliary transport in the trachea. In an ovine perfused in vitro tracheal model (*N* = 9) the epithelium was exposed to 25 L/min of flow, heated to lamb body temperature (38 °C) and fully saturated with water vapor as the control, followed by either room air (22 °C and 50% relative humidity) or nebulized solutions of NaCl 7% and mannitol 20% up to 1 min for a short duration, until mucociliary transport had visually changed. Mucus transport velocity (MTV) and cilia beat frequency (CBF) were continuously measured with video-microscopy.

**Results:**

Exposing the tracheal epithelium to air heated to body temperature and fully humidified had stable MTV 9.5 ± 1.1 mm/min and CBF 13.4 ± 0.6 Hz. When exposed to flow of room air, MTV slowed down to 0.1 ± 0.1 mm/min in 2.0 ± 0.4 s followed by a decrease in CBF to 6.7 ± 1.9 Hz, after 2.3 ± 0.8 s. Both MTV and CBF recovered to their initial state when heated and humidified air-flow was re-introduced. Exposing the tracheal epithelium to nebulized hypertonic saline and nebulized mannitol for 1 min increased MTV without a subsequent increase in CBF.

**Conclusions:**

This study demonstrates mucociliary transport can deteriorate within seconds of exposing the tracheal epithelium to flowing room air and increase rapidly when exposed to nebulized hypertonic solutions. The reduction in MTV precedes slowing of CBF with room air and MTV increases without a subsequent increase in CBF during the nebulization. Their relationship is non-linear and a minimum CBF of approximately 6 Hz is required for MTV > 0, while MTV can reach 10.9 mm/min without CBF increasing. Clinically these findings indicate a potential rapid detrimental effect of breathing with non-humidified air via bypassed upper airways and the short-term effects of nebulized osmotic agents that increase MTV.

## Background

During nasal breathing, room air is heated and humidified when passing through the upper airways [1] and becomes fully saturated with water vapor just below the carina [2, 3]. In spite of variable inspired air temperatures, humidities, and flows, the upper airways are able to heat and humidify incoming air due to the extensive vascular system in the nasal mucosa, which is capable of producing large and varying quantities of secretions [4]. Inadequate humidification of inspired air leads to impaired mucociliary transport in the tracheobronchial tree [5]. In clinical practice, spontaneously breathing tracheostomized patients are at a greater risk of inadequate humidification due to the bypassed upper airways. When nasal breathing, most heat and moisture exchanges between the air and airway surface occur in the nose; the tracheobronchial mucosa only adds 20% of the total water required for the relative humidity (RH) to reach 100% in the main bronchi [6]. In contrast, patients breathing room air through the stoma in the trachea forces the tracheobronchial epithelium to take over much of the air conditioning, causing the mucosa to have a greater water loss as it would need to add 85% of the total water required to achieve RH 100% in the main bronchi [6]. The increased water demand from tracheobronchial epithelium is not well compensated, which results in thick secretions and impaired mucociliary transport [7–11]. These symptoms are commonly found in patients with tracheostomy unless careful attention is given to heating and humidifying inspired air.

Williams et al. [12] proposed a model for the relationship between the airway mucosal dysfunction and temperature and humidity of the inspired gas. The authors suggested that respiratory gases at body temperature and RH 100% are optimal for mucociliary transport and even small changes to the temperature or humidity result in the negative impact on the mucociliary. Subsequent publications have supported this model, showing a deterioration in mucus transport velocity (MTV) and cilia beat frequency (CBF) after prolonged exposure (2 to 3 h) to air temperature and humidity below the conditioning provided by the upper airways [5, 13].

Osmotic agents are known to stimulate mucociliary transport through an osmotic effect on the airway surface, increasing the volume of the airway surface liquid and improving the rheologic properties of the mucus layer, resulting in accelerated mucus transport [14, 15]. Osmotically active substances used in respiratory care are nebulized hypertonic saline and less commonly mannitol in the dry powder formulation, both shown to improve mucus clearance in patients with airway diseases [16, 17]. Repeated nebulized 7% hypertonic saline inhalation is known to acutely enhance mucociliary clearance in tracheostomized patients [18] and in both healthy subjects and patients [17, 19]. Previous in vitro studies have reported a reduction in CBF with hypertonic saline immersion, where ciliostasis was observed within a few minutes of exposure to 3 and 7% saline solution [20]. Delivery of mannitol by inhalation as a dry powder has been reported to increase tracheal mucus velocity [21], improving mucus clearance due to an increase in osmolality of the mucus. Nebulized soluble mannitol has also been shown to increase mucociliary clearance [22] and improve pulmonary function [23], when compared with hypertonic saline.

Exposing the airways to cold, dry air and nebulized osmotic substances are known to influence mucociliary transport, but how quickly changes begin to occur is not fully understood [5, 13]. In this study, we tested the hypothesis that exposing the airway epithelium to flow of room air, that occurs during spontaneous breathing, will have a negative effect on mucociliary transport while nebulized hypertonic saline and mannitol solutions stimulate mucociliary transport. Publications to date have measured the effect of air temperature and humidity on either MTV or CBF over hourly time intervals [5, 13] and osmotic agents administration as soon as 5 min after exposure. However, there are no reports of MTV and CBF measurements during and immediately after the treatment period. In these experiments, we used a perfused in vitro tracheal model to continuously measure MTV and CBF, second-by-second, using video-microscopy to monitor changes in mucociliary transport when the tracheal epithelium was exposed to the flow of room air and nebulized hypertonic saline (7%) and mannitol solution (20%).

## Methods

### In vitro ovine model

**Fig. 1:**
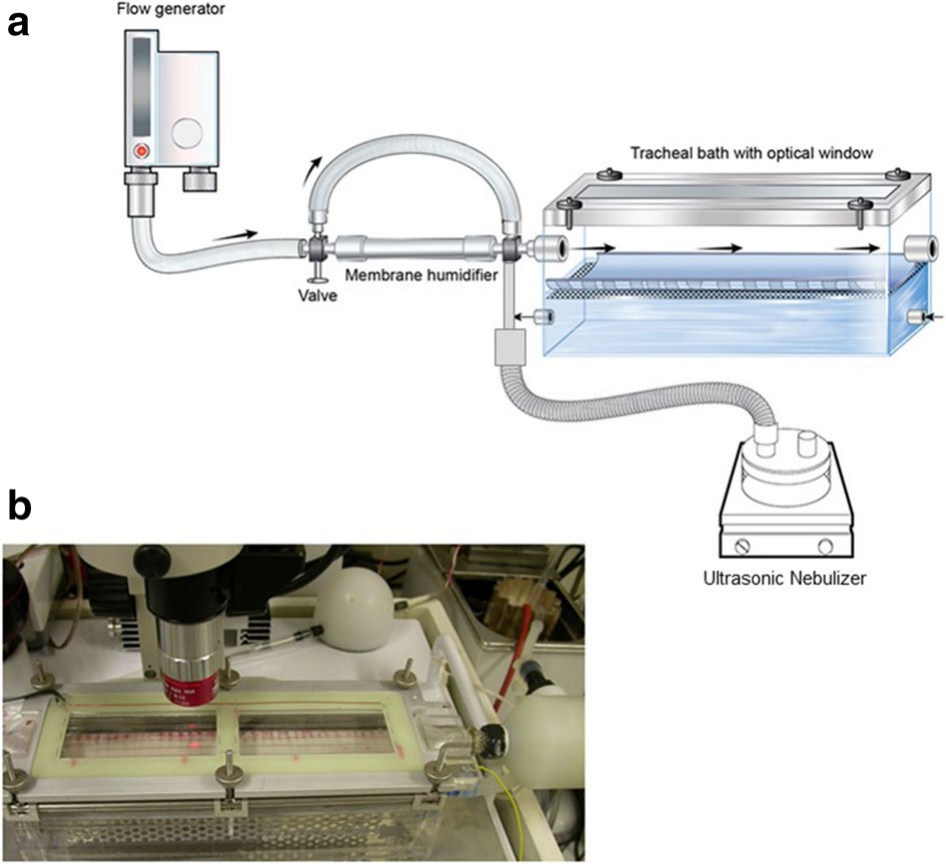
**a** Diagram of the experimental setup used for in vitro measurements of mucociliary transport on the tracheal epithelium of perfused lamb trachea. Air-flow was set to 25 L/min and was directed from left to right while mucociliary transport was from right to left. The ultrasonic nebulizer was introduced to the air path before the organ bath. Video-microscopy recordings were made through the heated optical window on the top of the tracheal bath to measure mucociliary transport along the tracheal epithelial surface. **b** Photo of the in vitro experiment setup with the trachea mounted in the organ bath, with circulation of Krebs–Henseleit solution below. The microscope with coaxial illumination is shown above

As described previously [13, 24] and briefly here, lamb tracheas (approximately 160 mm in length) were collected from a local abattoir immediately after slaughter and were transported to the laboratory. The tracheas were opened longitudinally along the ventral mid-line and fixed flat with the epithelium positioned upward in a custom-made bath placed on a vibration-proof table (Fig. 1). The opposite side of the trachea was bathed in recirculating Krebs–Henseleit solution (flow 50 mL/min, 2 L reservoir) at 38 °C that was oxygenated with carbogen gas. A flow generator was connected to a three-way valve, which allowed the air path to switch between a membrane humidifier or a bypass path before connecting to the tracheal bath. Unidirectional flow was maintained at 25 L/min with estimated velocity across the surface over the tracheal epithelium of ca. 0.4 m/s. The air was conditioned to 38 °C and RH 100%, passing through the Nafion™ (DuPont, USA) membrane humidifier (FC-125–240-5PP, Perma Pure, New Jersey, USA) with perfused and temperature-controlled deionized water for precise control of temperature and humidity of incoming air. Delivery of room air (22 °C and RH 50%) to the tracheal bath was achieved by switching the three-way valve to the bypassed airpath. Both sodium chloride and the mannitol solution were administered to the trachea by an ultrasonic nebulizer (Ultra- Neb 2000, DeVilbiss, Germany) connected to the air path of the organ bath, upstream, with air conditioned to 38 °C and 100% RH. The nebulized solutions were entrained into the conditioned airflow by connecting the humidifier to the inlet of the nebulization chamber. The top cover of the bath included a heated optical window with an anti-reflecting coating for video-microscopy.

### Video-microscopy

A video microscope (VMU-V, Mitutoyo, Japan) with a long working-distance lens (M Plan ApoNIR 5x, Mitutoyo, Japan) and in-line coaxial illumination was connected to a monochrome digital camera (Lm075, Lumenera, Canada), which recorded mucociliary transport at 60 frames per second over an area of 1.0 mm × 0.7 mm (640 × 480 pixels) (Additional file 1: Vides S1). During nebulization, video recordings were made with a digital camera (DCR-TRV33E, Sony, Japan) at 30 frames per second over an area of 920 µm × 615 µm (720 × 480 pixels) (Additional file 2: Video S2, Additional file 3: Video S3). The experiments were performed 1 h after the tissues were mounted to allow dissipation of any released mediators [13].

### Temperature and humidity settings

The effect of a change in the air temperature and humidity on mucociliary transport was observed in 7 tracheas. Video-microscopy recording were made for the duration of each experiment. The experiments started with the air temperature and humidity set to body temperature of lambs (38 °C) and RH 100% before intermittent exposure to 25 L/min flow of room air at 22 °C and RH 50% until the movement of particles across the video-microscope field-of-view had stopped, after which the humidified air-flow was returned.

### Nebulized hypertonic saline

The effect of nebulized hypertonic saline (7% NaCl) and mannitol solution (20%) for 60 s, entrained with air conditioned to 38 °C and RH 100%, was observed on mucociliary transport in the trachea with video-microscopy recordings made for the duration of the experiment. Both saline and mannitol solutions were made using purified water (Milli-Q, Merck & Co, USA).

### Video-microscopy analysis

**Fig. 2:**
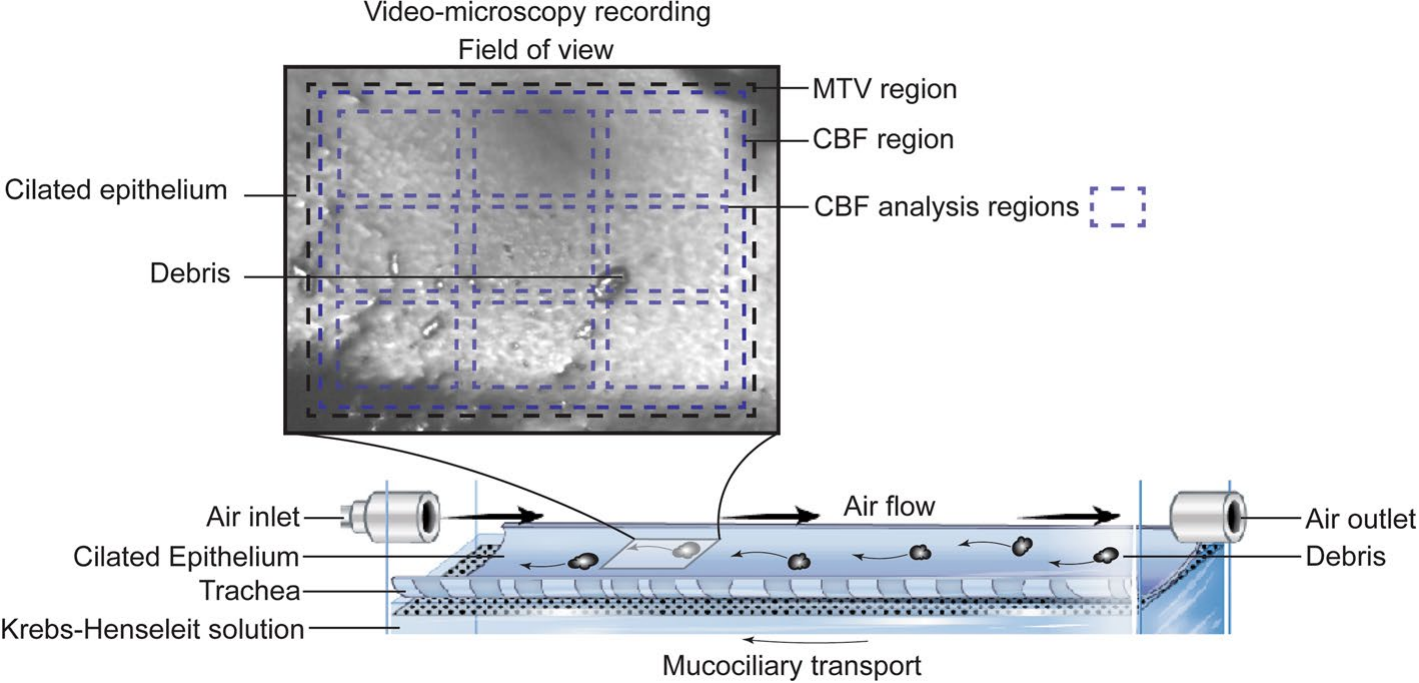
Field of view from video-microscopy recordings and the analysis region for the mucus transport velocity (MTV) and cilia beat frequency (CBF) with the 3-by-3 gridded subset of regions for more detailed analysis. Arrows indicate the direction of air-flow and mucociliary transport

MTV was determined from the recordings by tracking the natural visible particles on the surface of the mucus as they moved across the field-of-view. CBF was determined using Fourier analysis of the video-microscopy recordings with a rolling 2.1 s window and curve fitting to locate frequency peaks using MATLAB (MathWorks, USA, see Additional file 4). CBF was measured in ten analysis regions: one region covering most of the field-of-view and nine smaller regions, arranged in a 3-by-3 grid (Fig. 2). These smaller regions were used to measure variation in CBF over the field-of-view. For the hypertonic saline treatment only, CBF was measured over the entire field-of-view from the first 35 s followed by a single region in the top left corner of the video; an abundance of debris prevented robust measurement in other portions of the frame. The relative area, within the field-of-view, where cilia activity was visible during mucociliary transport and after mucociliary transport had ceased was calculated from the recordings.

### Data analysis

Measurements are presented as the mean ± standard deviation and were compiled and analyzed in Excel (Microsoft, USA) and GraphPad Prism 5 software (Prism, USA). Differences were considered statistically significant when a two-tailed t-test produced a *p* < 0.05.

## Results

**Fig. 3:**
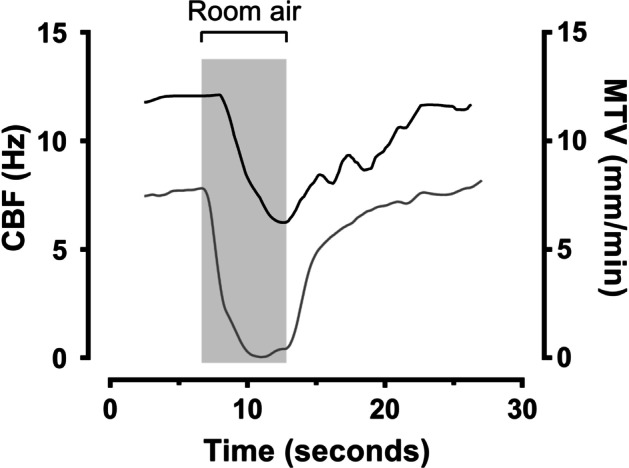
An example of the effect of intermittent exposure of room air on the cilia beat frequency (CBF) and mucus transport velocity (MTV) from a video-microscopy recording shown in Additional file 1: Video S1. The gray region represents room air and white regions represent air at body temperature and fully saturated with water vapor

Video-microscopy recordings of the tracheal epithelium show mucociliary transport moving particles across the field-of-view, from which MTV was obtained, and beating cilia as a background flicker, from which CBF was obtained (Additional file 1: Video S1). A notable reduction in the particle speed and the background flicker is observed when the tracheal epithelium was exposed to the flow of room air. Short room air exposure times were sufficient to stop mucus transport but not long enough to cause any irreversible damage to mucociliary transport, seen as the MTV and CBF recovering to initial values when air was returned to body temperature (38 °C) and RH 100% (Fig. 3).

**Fig. 4:**
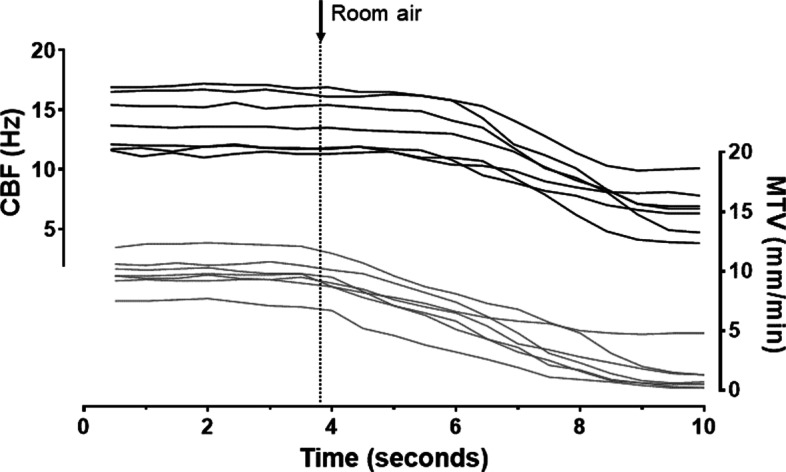
Cilia beat frequency (CBF) (black) and mucus transport velocity (MTV) (gray) measured over time. The dotted vertical line marks the change in air passing above the surface from body temperature and fully saturated with water vapor (38 °C and 100% relative humidity) to room air (22 °C and 50% relative humidity)

**Fig. 5:**
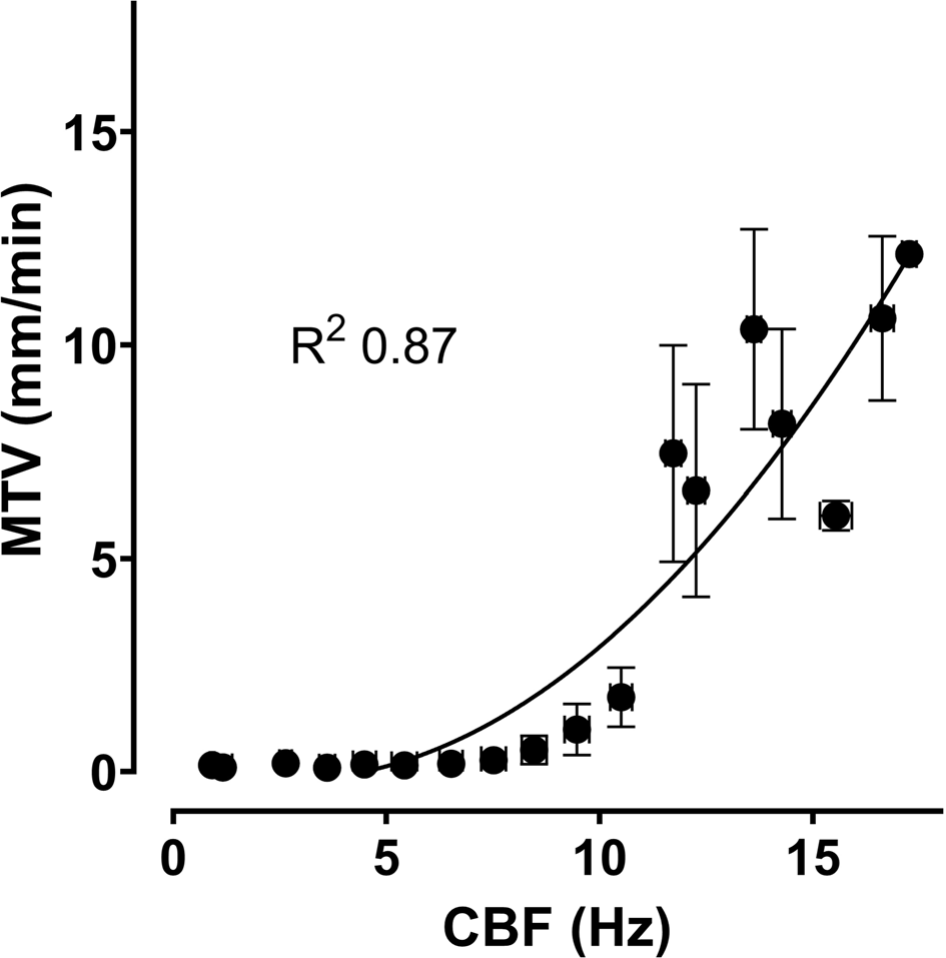
Correlation between overall mean mucus transport velocity (MTV) and cilia beat frequency (CBF) measurements under different air temperatures and humidities. Non-linear regression gives an R^2^ of 0.87 and suggests that a minimum CBF of ca. 6 Hz is required for MTV > 0

When the air temperature and humidity were decreased by exposing the tracheal epithelium to room air, the MTV changed almost immediately, while the CBF started changing after a 2.3 ± 0.8 s delay and did not stop completely before heated and humidified air was restored (Fig. 4). The changes in MTV and CBF caused by the exposure to room air resulted in significant decreases in MTV, which dropped from 9.5 ± 1.1 mm/min to 0.1 ± 0.1 mm/min (p < 0.05) in 2.0 ± 0.4 s and in CBF, which dropped from 13.4 ± 0.6 Hz to 6.7 ± 1.9 Hz (p < 0.05) in 3.7 ± 0.6 s (Table 1). The changes in MTV and CBF were highly correlated (R^2^ = 0.87, Fig. 5) using a second-order polynomial model.

**Table 1:** Mean mucus transport velocity (MTV) and cilia beat frequency (CBF) measurements under body temperature and fully saturated with water vapor (38 °C and relative humidity (RH)100%) and room air (22 °C and RH 50%) from each analyzed video

Sample	Mucus transport velocity (mm/min)			Cilia beat frequency (Hz)			Delay (s)
	38 °C and RH 100%	22 °C and RH 50%	Time (s)	38 °C and RH 100%	22 °C and RH 50%	Time (s)	
1	7.6 ± 0.1	0.2 ± 0.1	2.0	11.9 ± 0.2	6.3 ± 0.0	3.6	1.6
2	9.2 ± 0.2	4.8 ± 0.7	2.2	11.7 ± 0.1	8.0 ± 0.2	4.9	2.7
3	12.2 ± 0.1	1.3 ± 0.3	1.9	17.1 ± 0.2	10.3 ± 0.2	3.8	1.9
4	10.6 ± 0.4	0.7 ± 0.5	1.4	11.4 ± 0.1	3.8 ± 0.0	2.7	1.3
5	9.8 ± 0.7	0.2 ± 0.1	2.6	16.7 ± 0.5	6.9 ± 0.0	3.5	0.9
6	10.2 ± 0.5	0.5 ± 0.4	2.4	15.3 ± 0.1	4.7 ± 0.1	4.1	1.7
7	9.6 ± 0.3	1.3 ± 0.8	1.7	13.6 ± 0.2	6.7 ± 0.0	3.6	1.9

Results are presented as the mean ± standard deviation. Time measurements represent the time the MTV and CBF took to change from measurements made at 38 °C and 100% RH to measurements made at 22 °C and RH 100%. The delay is the difference between the MTV and the CBF times taken for change

**Fig. 6:**
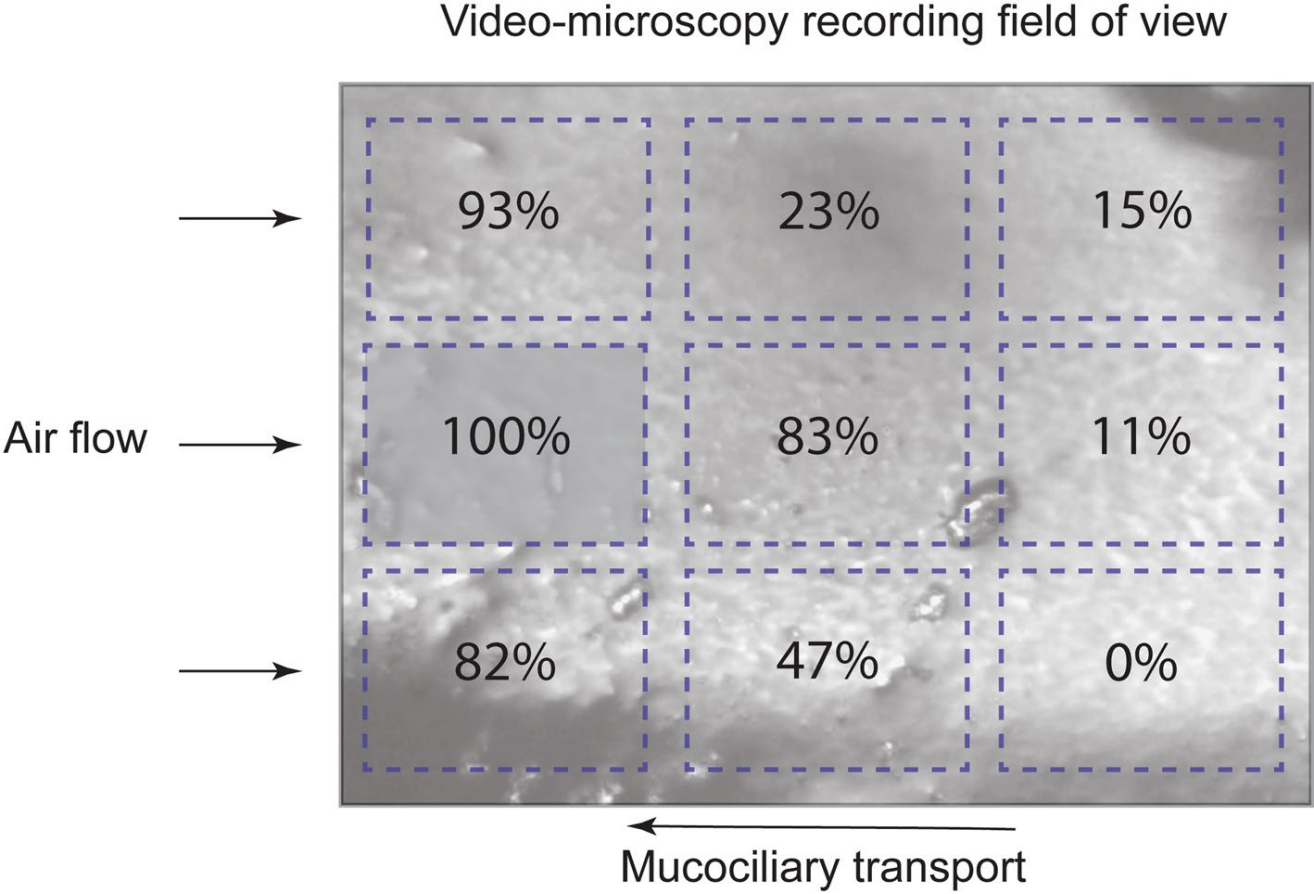
The average percentage change in cilia beat frequency, from all video-microscopy recordings, made when the tracheal epithelium was exposed to room air (22 °C and 60% relative humidity) where 100% represents the largest percentage change

Variability in CBF was observed visually and measured in the nine regions spread across the field-of-view (Fig. 6). This showed that the greatest change in CBF (> 80%) in regions closest to the air-flow inlet (left side of the field-of-view) when air temperature and humidity were changed.

**Fig. 7:**
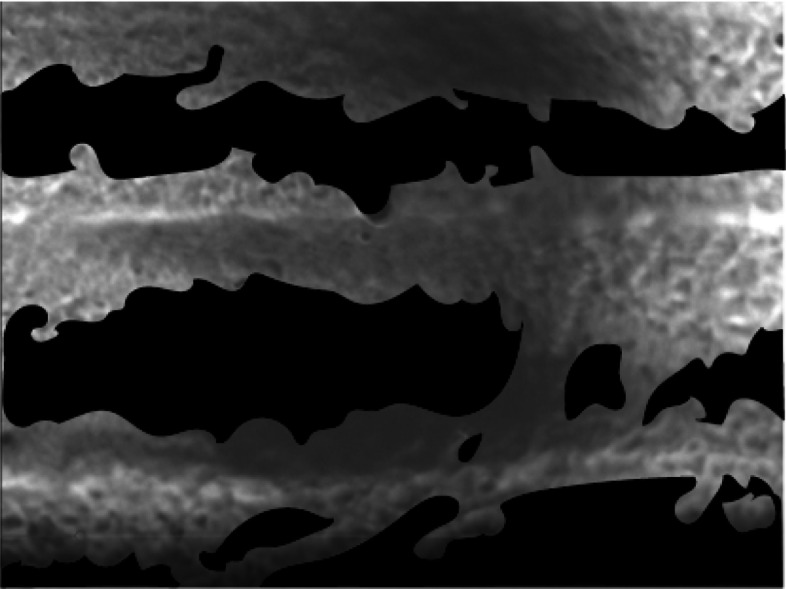
Representative image showing regions (white) where cilia activity continued (63% of the area) after mucociliary transport had ceased shown in Additional file 1: Video S1

Cilia activity was observed in approximately 60% of the field-of-view when mucociliary transport had ceased (MTV = 0), though cilia activity continued in as much as 93% in one sample and was as low as 28% in another. The regions where cilia remained active were typically longitudinally aligned with the perfused tracheal tissue (Fig. 7). Cilia activity was visible in 89% to 100% of the video frame when the cilia were exposed to heated and humidified air. The remaining parts of the video were too dark to discern if cilia activity was present, possibly because of the contours of the tissue reducing reflection of light during a coaxial illumination.

**Fig. 8:**
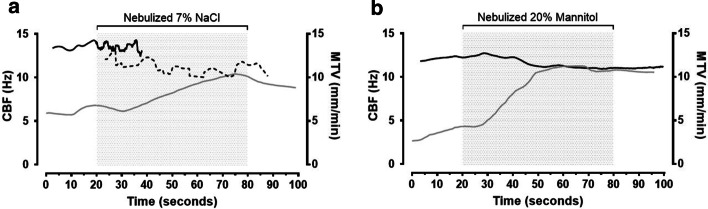
An example of the effect of nebulized hypertonic saline (7% NaCl) (**a**) and nebulized mannitol (20% Mannitol) (**b**) on the mean cilia beat frequency (CBF) (black) and mucus transport velocity (MTV) (grey) across the field-of-view from a video-microscopy recording shown in Additional file 2: Video S2, Additional file 3: Video S3. The shaded area represents nebulized 7% NaCl in **a** and nebulized 20% mannitol in **b**. Different regions were used for CBF analysis in **a** shown as a solid black line for measurements made over the entire field-of-view and a dashed line showing measurements made in a sub-region in the top left corner or the recording, since an abundance of debris prevented robust measurement in other portions of the frame

Both nebulized hypertonic saline and mannitol caused a similar response in mucociliary transport on the tracheal epithelium, when entrained with air conditioned to 38 °C and RH 100%. Nebulized hypertonic saline resulted in an increase in MTV followed by a decline in CBF (Fig. 8, Table 2). Nebulized mannitol also caused MTV to increase while CBF decreased over this period. The decline in CBF was not as marked with mannitol (2.3 Hz) compared to hypertonic saline (5.5 Hz).

**Table 2:** Mean mucus transport velocity (MTV) and cilia beat frequency (CBF) measured under body temperature (38 °C) and fully saturated with water vapor (100% relative humidity) (Baseline) and following nebulization with 7% NaCl or 20% mannitol, entrained with the same air conditions

Nebulized solution	Mucus transport velocity (mm/min)		Cilia beat frequency (Hz)	
	Baseline	Following nebulization	Baseline	Following nebulization
7% NaCl	6.1 ± 0.4	8.3 ± 1.5	13.5 ± 0.3	8.0 ± 4.1
20% Mannitol	3.5 ± 0.5	8.8 ± 2.6	13.5 ± 0.3	11.2 ± 0.6

Results are presented as the mean ± standard deviation

## Discussion

Analysis of video-microscopy recordings made of the tracheal epithelium surface enabled simultaneous measurements of MTV and CBF and the ability to observe their rapid changes when exposed to a drop-in air temperature and humidity. The presented results show both MTV and CBF decrease quickly when the epithelium is exposed to room air at 25 L/min, a flow comparable to that observed during normal breathing [25]. These changes were reversible when the epithelium was exposed to air heated to body temperature and fully saturated with water vapor. Irreversible changes to mucociliary transport on the tracheal epithelium have been reported after 180 min of exposed to air cooler than body temperature and fully saturated with water (30℃, RH 100% and 34℃ RH 100%) [13] and during ventilation in vivo with air at 23℃ and RH < 10% [5]. The strong correlation between MTV and CBF when the trachea was exposed to heated and humidified air is consistent with a causal relationship, but the delayed change in CBF compared to MTV when exposed to room air (22 °C and RH 60%) suggests that the slowing of MTV was not initiated by a reduction of CBF, but rather by changes to the mucus properties. Further, the increase in MTV seen when exposed to nebulized hypertonic saline and mannitol, with a decline in CBF, also suggest that it is the composition of the airway surface liquid that determines mucociliary transport, rather than the frequency of beating cilia.

Although our data show MTV and CBF to be highly correlated under stable conditions, MTV was found to change more quickly than CBF when the epithelium was exposed to room air. Kilgour et al. 2004 [13] reported a similar result when the tracheal epithelium was exposed to low air temperatures for a prolonged period; the CBF had a longer survival time compared to the MTV. Cold, dry air changes mucus properties, such as viscosity, and causes changes to cilia structure and coordination [11, 26, 27]. The initial decrease in MTV reported in the study could be attributed to changes in mucus properties and the subsequent slowing of CBF. During exposure to nebulized hypertonic saline and mannitol, the initial increase in MTV could also be attributed to changes in mucus properties, due to osmotic activity, facilitating greater MTV without an increase in CBF. Mucus properties are known to be important for maintaining effective mucus–cilia interactions, required for MTV to clear debris from the airway [28, 29]. Mucus is a hydrogel and acts as a protective layer where it maintains hydration and prevents desiccation of the ciliated epithelium cells [30]. The mucus layer is in direct contact with air passing through the conducting airways and is the first to respond to changes in air conditions. When inspired air is colder and dryer, the thermal imbalance forces heat and moisture transfers from the mucus surface into the air. This removes water from the mucus layer by evaporation, which in turn lowers the surface temperature, and ultimately causes the mucus to become more viscous [31, 32]. Mucus with greater viscosity may interfere with the cilia’s ability to propel it along the surface, which can result in mucus accumulation and the need for suctioning in patients with tracheostomy [7]. With prolonged exposure, the protective function of the mucus layer lessens and thermal changes would begin to affect the beating cilia underneath, causing cilia activity to decline [13]. Cilia are known to be affected by temperature [33] and their discoordination affects mucociliary transport [34]. The thermodynamic balance between latent and sensible heat transfer during normal inspiration and exhalation, which infers water movement in and out of the mucus as a result of different temperature and water vapor content in air, needs to be investigated in vivo.

In the time scales considered in this study, the decrease in MTV appears to precede the slowing of the CBF and the increase in MTV with nebulized hypertonic saline and mannitol precedes any change in CBF; these effects are expected to occur outside of the recorded field-of-view. As viscosity increases, the cilia’s combined force is no longer sufficient to propel the mucus layer. Mucus is a non-Newtonian fluid [32] and its viscosity decreases under shear forces introduced by the beating cilia. It is also possible that, due to water losses with exposure to room air, the mucus becomes more viscous, leading to a reduction in CBF that slows MTV. A visual inspection of the video-microscopy recordings revealed the debris on the top of the mucus layer went out of focus when MTV slowed down. This change in the recording’s focus suggested the airway surface liquid, including the periciliary layer, receded, caused by the evaporation of water when the tracheal epithelium was exposed to flowing room air. On the other hand, when mucus becomes more hydrated, following the effect of osmotically active substances, the viscosity should decrease, resulting in an increase in MTV. It appears that the increased osmolarity in the airway surface liquid does not have the same effect on CBF, which, instead, suppresses ciliary activity. With nebulized hypertonic saline, the CBF on the tracheal epithelium decreased during exposure; while with nebulized mannitol, the CBF remained relatively unchanged. Since hypertonic saline is ionic and charged, it can rapidly permeate the ciliated epithelial cell membranes. Contrary to saline, mannitol is a sugar and it is ion free with a low permeability index. This allows it to provide an osmotic effect on the mucosa without permeating the ciliated epithelium, allowing ciliary function to continue [35]. The difference in permeability of the nebulized solutions could be the reason different effects are seen in CBF while stimulating MTV.

Another potential mechanism that may reduce CBF when exposed to cooler dryer air is from the cooling of the airway epithelium. The lower temperature of room air and the evaporation of water from the epithelium cause the heat losses which decrease the temperature of the ciliated epithelium, slowing biochemical reactions in microtubules of the motile cilia and result in reduced CBF. It is known that, to beat, cilia require ATP produced by enzymes that are temperature dependent [33]. Jones et al. [36] estimate that evaporation from the mucus surface leads to a 2 to 3 ℃ change of surface temperature in the nasopharynx during quiet breathing with room air. In addition, Smith et al. [37] showed that nasal cilia continue to beat with a normal pattern at temperatures as low as 2 ℃. Although the authors did not measure the surface temperature of the tracheal epithelium, and the above-mentioned reports were measured in the nose, known to tolerate different air temperatures [4], it is unlikely that the cilia in the tracheal epithelium measured in this study were cooled below 2 ℃. This suggests that a change in physical properties of the airway surface liquid, from rapid dehydration and an increase in viscosity, caused CBF to slow down.

Regional variation in CBF could be caused by the variability in mucus thickness and non-homogeneous mucus properties which occur naturally on the trachea’s surface [38]. Variability in mucus properties results from proximity to secretory cells and surface contours which create streams and plaques of mucus movement [39]. The evaporation or addition of water and the subsequent changes of mucus viscosity and MTV are likely to be slower in regions where the mucus layer is thicker, or partly protected by contours in the tissue, allowing patches of cilia activity to continue. This can be seen in the video-microscopy recordings as areas where cilia continue to beat, after exposure to room air or nebulized hypertonic solutions, while appearing stationary in others.

Limitations of this study include a lack of humidity measurements of the air above the trachea and the temperature on the surface of the tracheal epithelium. While this information would be useful for demonstrating how quickly room air begins to affect mucociliary transport on the tracheal surface thermodynamically, the intent of these experiments was to present changes in tracheal mucociliary transport when exposed to the flow of room air (25 L/min) that could occur during breathing through a tracheostomy, without humidified inspired air. The flow profiles usually found in normal breathing show that the maximum inspiratory flow is only reached over a short period; however, in this experiment design the authors used a constant flow, comparable to this maximum only experienced intermittently during normal breathing, to observe the effects on mucociliary transport. In addition, the flow in the experiment design was unidirectional, preventing the heat and moisture recovery during exhalation in tidal breathing. This design enabled the authors to assess mucociliary transport for analyses of the transition period and it negated any confounding effects from ventilation with varying respiratory rates and tidal volumes that may induce shear stress, which is known to influence mucociliary transport [24, 40]. Additionally, the experiment on the effects of nebulized hypertonic saline introduced a significant artifact into the field-of-view from the aerosols. Due to the difference in density and viscosity of saline and mannitol solutions, the delivered amount of each aerosol is likely to be varied. The osmolarity of 7% NaCl and 20% mannitol are also different and, as such, they produced a different effect on MTV and CBF, however, comparing the efficacy of these two osmotic agents was outside of the scope of this study.

Many clinical questions related to time of exposure of the epithelium to respiratory gases with different levels of humidification need to be assessed during tidal breathing. We did not study physical properties of mucus because of the short duration of exposure of the epithelium to room air and the complexity of collection of mucus in very small quantities. Also, due to the limited resolution of the video-microscopy recordings the authors were unable to look closely into the cilia coordination on very short time scales. The opposing effects of dry air and nebulized osmotic agents’ exposure on the mucociliary transport along the tracheal epithelium were not studied in combination here. The chamber used to house the perfused trachea had a rectangular cross-section for the air path when it passed over the epithelium, which may have produced a different velocity profile to that found in the cylindrical trachea. This difference in flow speed, mixing and eddy could also have an effect on the heat and moisture exchange between the epithelium and air.

It is unlikely that the circulating Krebs–Henseleit solutions on the outer surface of the trachea could substitute blood microcirculation, which maintains the heat and moisture on the epithelial surface in vivo. Therefore, extrapolating the presented results to the clinical condition should be performed with caution as the effects reported in the study could represent an extreme. However, these in vitro experiments demonstrate high sensitivity of the tracheal epithelium to changes in temperature and humidity induced by flow of room air over the tracheal epithelium over few seconds. The long-term effects of cold and dry air on mucociliary transport and the airway epithelium require an in vivo setting to reproduce the complex thermodynamic and physiological mechanisms that maintain water content in the airway surface liquid during breathing.

## Conclusion

This study demonstrates that mucociliary transport can deteriorate within seconds of exposure to the flow of room air and quickly accelerate during nebulization of hypertonic saline and mannitol solutions. The reduction in MTV precedes the slowing of the CBF when exposed to room air, which suggests that, at least initially, it is a change in physical properties of the airway surface liquid that affects the mucus–cilia interactions. The increase of MTV, during nebulization of the osmotic agents, while CBF decreases or remains relatively unchanged, further suggests that changes in the properties of the airway surface liquid control mucociliary transport. These findings indicate that exposing conducting airways to the flow of room air can rapidly impair mucociliary transport, which could potentially result in mucostasis with a risk of airway obstruction. Patients with tracheostomy would be at greatest risk as the as the conditioning upper airways are bypassed. The nebulized osmotic agents, at least in the short-term, increase MTV, but they may also impair the ciliated epithelium in the longer term or with higher dose that requires further research.

## Supplementary Information

**Additional file 1: Video S1.** Video-microscopy recording of mucociliary transport in vitro (left) when exposed to room air (22 °C and 50% relative humidity) at *t* = 4–12 s and *t* = 29–34 s and returned to body temperature and fully saturated with water vapor (38 °C and 100% relative humidity) in between. The recording demonstrates repeated exposure of room air on the tracheal epithelium. The corresponding mucociliary transport measurements of cilia beat frequency (CBF) and mucus transport velocity (MTV) are plotted on the right.

**Additional file 2: Video S2.** Video-microscopy recording of mucociliary transport in vitro (left) when exposed to air heated to body temperature and fully saturated with water vapor (38 °C and 100% relative humidity) for 20 s followed by entrained nebulized hypertonic saline for 60 s with the same air conditions. The corresponding mucociliary transport measurements of cilia beat frequency (CBF) and mucus transport velocity (MTV) are plotted on the right. The CBF measurements were made using different regions of the frame due to an abundance of debris prevented robust measurement in other portions of the frame.

**Additional file 3: Video S3.** Video-microscopy recording of mucociliary transport in vitro (left) when exposed to air heated to body temperature and fully saturated with water vapor (38 °C and 100% relative humidity) for 20 s followed by entrained nebulized mannitol solution for 60 s with the same air conditions. The corresponding mucociliary transport measurements of cilia beat frequency (CBF) and mucus transport velocity (MTV) are plotted on the right.

**Additional file 4.**

## Data Availability

Data available on request.

## Abbreviations

CBF: Cilia beat frequency
MTV: Mucus transport velocity
RH: Relative humidity
