# Supplementary material for: Rapid changes in mucociliary transport in the tracheal epithelium caused by unconditioned room air or nebulized hypertonic saline and mannitol are not determined by frequency of beating cilia
Source: Intensive Care Med Exp. 2021 Mar 17;9:8. doi: 10.1186/s40635-021-00374-y (PMC7966670; doi:10.1186/s40635-021-00374-y)
Supplement: Supplementary file 4 — Additional file 4. [file 40635_2021_374_MOESM4_ESM.docx]

**Supplemental Material: A detailed method for cilia beat frequency analysis from video-microscopy recordings.**

# Introduction

Beating cilia were recorded at 60 frames/second using a video-microscope. The frequency of cilia activity (cilia beat frequency, CBF) was determined from the video-microscope recordings using Fourier analysis in Matlab (The MathWorks Inc, MA). The data analysis was broken into two parts:

1. extraction of frequency content in the video, and
2. peak-frequency estimate.

All signals can be represented with sums of sinusoids parameterized by amplitude, frequency and phase. Frequency content extraction determined the amplitude of each frequency component present in the video-microscopy recording. These frequencies contain information about not only beating cilia, but also debris moving across the field-of-view, sample movement and temporally varying illumination. The CBF was determined by selecting the highest peak in the amplitude of the frequency content extracted from the video.

# Analysis regions

The analysis was generally completed for ten regions across the field-of-view captured by the video-microscopy recordings (Figure. 1, for example). The location of these CBF analysis regions varied slightly between videos, however, in each case the first region spanned most of the frame (providing an ‘average’ CBF). The remaining nine regions were all equal in size, non-overlapping and spread throughout the frame, as shown in the example (Figure 1). A CBF time-series was calculated for each region in the video. Analysing regions, rather than individual pixels, helped improve the signal-to-noise ratio and reduce memory required for the analysis. Breaking the field-of-view into multiple regions provided granularity to investigate variation in CBF across the region of tissue observed. The region position did not generally affect the calculated frequency content unless there was no activity across most of the region (if the sample was dark, for example).

**Figure 1.** Typical CBF analysis regions processed to calculate cilia beat frequency.

#

# Frequency content extraction

A time-series for the frequency content of each region through the duration of the video was calculated using a rolling window. This involved gathering the image intensity time-series for every pixel in each region over 128 frames (approximately 2.1 seconds) and calculating the fast-Fourier-transform on each time series separately (e-Figure. 2). The amplitude of the fast-Fourier-transform was averaged for every pixel in the window to reduce noise and produce a slice of the frequency content for the region for the 2.1 second duration window. These individual slices are stacked together as the window rolls through the video, one frame at a time, producing a three-dimensional (time, frequency and amplitude) dataset. This data can be examined using a waterfall plot (Fig. 3), which maps amplitude to colour and illustrates the most active frequencies as time progresses through the video recording.


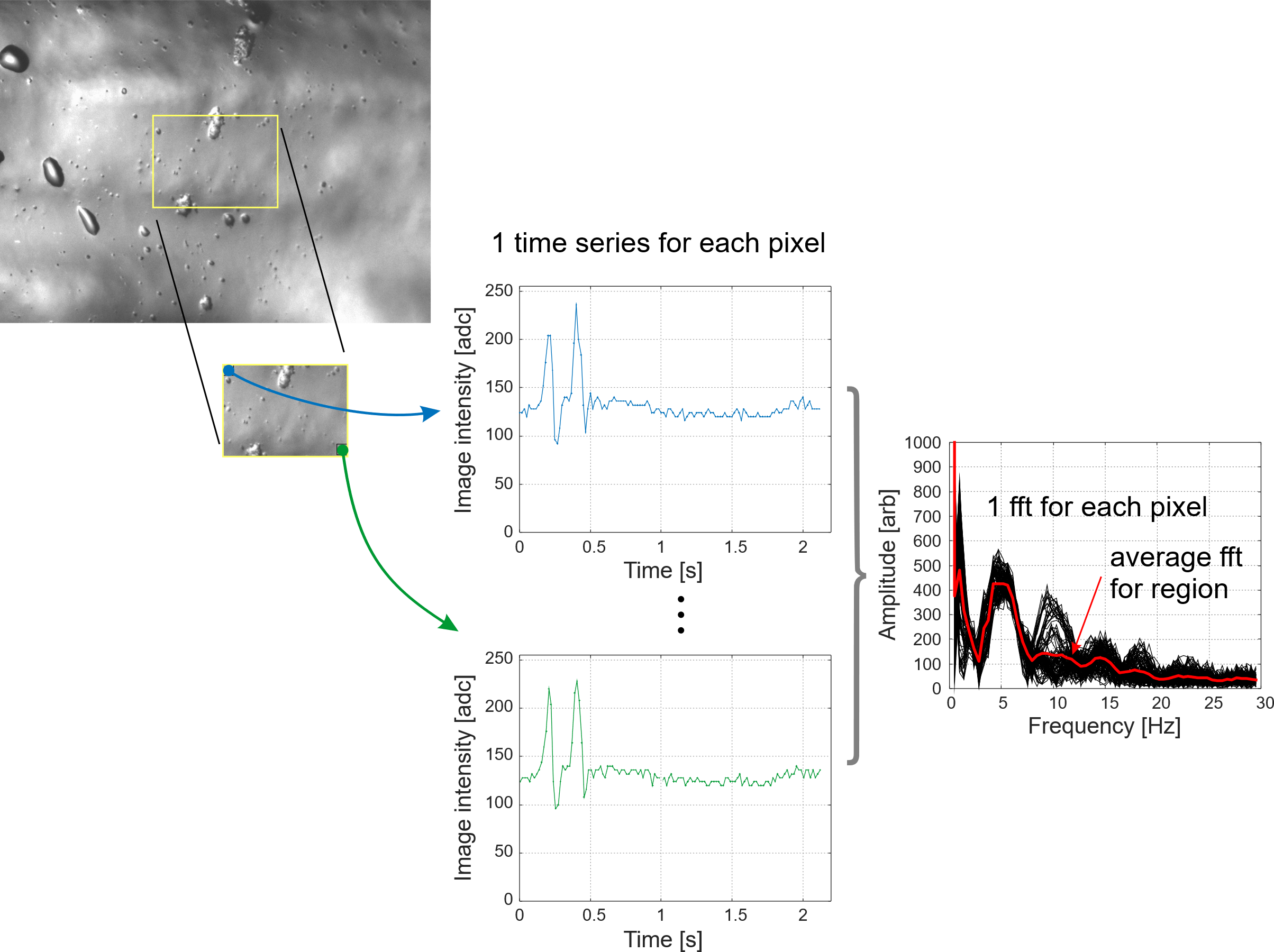


**Figure 2.** The average frequency content is calculated using fast-Fourier transform (fft) from the time series of each pixel within the region over approximately 2.1 seconds, the length of the rolling window.

**Figure 3.** A waterfall plot, from region 6, which combines frequency content from the rolling window passing through the video. The most active frequencies in each 2.1 second window are col­ored red and least active colored green and cyan. The first 127 frames (approximately 2 seconds) are shown in dark blue (missing data) because 128 frames are required to calculate the frequency content for each time step consolidated for this plot.

#

# Peak frequency estimate

The only periodic signal expected in the video is beating cilia, so the peak frequency is found in each window as an estimate of the CBF. The signal is generally not a simple peak and it is distorted by low frequency components, which arise from general illumination, sample movement and debris drifting through the region. Locating the peak frequency with a single peak function was prone to miss-fitting, particularly as the CBF reduced when the sample was exposed to room-air. Several model functions were explored. The most robust modelled the amplitude of the low-frequency components as a combination of decaying exponentials and the peak frequency using a Lorentzian curve:

$$A\left( f \right)=\left( c_{1}\cdot e^{-\frac{f}{c_{2}}}+c_{4}\cdot e^{-\frac{f-c_{5}}{c_{6}}}+c_{3} \right)+\left( \frac{h}{\pi\cdot\omega}\cdot\frac{\omega^{2}}{\left( f-f_{0} \right)^{2}+\omega^{2}}+h_{0} \right)$$

Here, *c_x_, h, ω, f_0_,* and *h_0_* are fitted coefficients and *f* is frequency. Fitting used Matlab’s fminsearch function to minimize an error function. The model was fitted in two parts. The first error function was the sum of squared difference between the pair of decaying exponentials and the frequency profile calculated for each window, as described above. The second error function was the sum of squared differences between the Lorentzian curve and the residuals of the first fit. These parts are grouped by parentheses in the equation above and illustrated in Figure 4.

**Figure 4.** Example frequency profile is plotted (blue) along with the components of the model used to locate the peak frequency (dotted: exponential component; dashed: Lorentzian component) and the complete model fit (red). This is the frequency content from frames 473 to 600 (the 128 frame window ending at 10 seconds in Figure 3).

Curve fitting was very sensitive to the initial parameter estimates provided to fminsearch, and the range of parameter values permitted. The initial values and constraints on the parameters are shown in Table 1. Only frequencies greater than 0.5 Hz were considered when fitting the dual-exponential component. The initial peak-frequency was estimated by placing line-segments on the waterfall plot manually. Linear interpolation of these line-segments was used to select a range of frequencies to fit the Lorentzian curve component. Frequencies within 2.5 Hz of the estimated peak were considered when fitting the Lorentzian curves to the residuals from the exponential decay model. This reduced the influence of the global illumination and debris passing through the frame.

Noise, from debris passing through the region often perturbed or prevented robust fitting. This could cause obviously erroneous fits to peak frequency or failure to fit at all. A 29-point median-filter (approximately 0.5 seconds) was applied to the time-series of the peak frequency fitted to each of the windows through the video.

**Table 1.** Initial parameter values and constraints applied during model fitting.

| Parameter | Initial | Constraint |
| --- | --- | --- |
| *c_1_* | Difference between maximum and minimum profile value (*height*). | none. |
| *c_2_* | 7 Hz | none. |
| *c_3_* | Minimum profile value. | none. |
| *c_4_* | *height*/4 | none. |
| *c_5_* | 0 Hz | none. |
| *c_6_* | 20 Hz | none. |
| *h* | Difference between maximum and minimum residual value (*height*). | 0 < h < 1000 |
| *ω* | 2 | 1.5 < *ω*< 20 |
| *f_0_* | Interpolated from manually placed guides (see text). | 0 < *f_0_*< 20 Hz |
| *h_0_* | Minimum residual value. | none. |

Several other approaches for finding the peak frequency were explored. However, there was not a great deal of difference in the results produced by these different methods. The approaches explored included:

- fitting Gaussian curves instead of Lorentzian curves,
- including three frequency profiles in the data used for each fit,
- fitting combinations of Lorentzian curves and decaying exponential curves,
- varying the range of frequencies considered during fitting,
- varying the constraints applied to parameter values during fitting,
- moving regions to avoid debris and dark spots in the video, and
- varying the initial conditions used in fitting.

Providing guides for the initial estimate of the peak frequency provided the greatest benefit. Without these guides, the model was more likely to choose quite different peak frequencies in adjacent windows. The guides did not eliminate this problem completely as noise in the frequency profile and the small amplitude of CBF peaks all complicated the fit.

# Selected source code

Matlab function used to extract the frequency content from regions in a video stream.

function Result = ExtractFrequencyContent(strFilename, ROIs, nWindow)

nRegions = size(ROIs,1);

oVideo = VideoReader(strFilename);

Result.FrameRate = oVideo.FrameRate;

Result.ROIs = ROIs;

Result.FrameCount = floor(oVideo.FrameRate * oVideo.Duration);

Result.Frequency = (0:(nWindow/2-1)) * Result.FrameRate/nWindow;

Result.Time = ((1:Result.FrameCount) - 1) / Result.FrameRate;

% Initialize

for iRegion = 1:nRegions

roi = ROIs(iRegion,:);

Result.History{iRegion} = zeros(roi(4) - roi(3) + 1, roi(2) - roi(1) + 1, nWindow);

Result.FrequencyData{iRegion} = zeros(Result.FrameCount, length(Result.Frequency));

end

% Fill the start of the buffers.

for iFrame = 1:(nWindow - 1)

frame = oVideo.readFrame();

for iRegion = 1:nRegions

roi = ROIs(iRegion,:);

Result.History{iRegion}(:,:,iFrame) = frame(roi(3):roi(4), roi(1):roi(2),1);

end

end

% Include a frame in the result for illustration purposes.

Result.AFrame = frame;

fprintf('Processing %s, %d frames, %.2f frames/second\n', strFilename, Result.FrameCount, Result.FrameRate);

% Start doing frequency analysis.

for iFrame = nWindow:Result.FrameCount

if rem(iFrame,100) == 0

fprintf('%d\n', iFrame);

else

fprintf('.');

end

if oVideo.hasFrame == 0

fprintf('Ran out of frames at frame %d!!\n', iFrame);

break;

end

frame = oVideo.readFrame();

for iRegion = 1:nRegions

roi = ROIs(iRegion,:);

Result.History{iRegion}(:,:,nWindow) = frame(roi(3):roi(4), roi(1):roi(2),1);

sz = size(Result.History{iRegion});

dd = reshape(Result.History{iRegion}, sz(1) * sz(2), sz(3));

%dc = mean(dd, 2);

%dd = dd - (dc * ones(1, sz(3)));

fft_dd = abs(fft(dd'));

mfft = median(fft_dd,2)';

Result.FrequencyData{iRegion}(iFrame,:) = mfft(1:length(Result.Frequency));

% Shuffle history.

Result.History{iRegion}(:,:,1:(nWindow-1)) = Result.History{iRegion}(:,:,2:nWindow);

end

end

Matlab function used to fit a dual-exponential decay curve. The function is supplied with the frequency scale (f) and frequency profile amplitude (A). function model = Fit_ExpDecayDual(f, A)

% Fit to the supplied data

sel = 0.5 <= f;

Freq_Subset = f(sel);

Ampl_Subset = A(sel);

Height = max(Ampl_Subset) - min(Ampl_Subset);

x0 = [Height, 7, min(Ampl_Subset), Height/4, 0, 20];

opts = optimset('MaxFunEvals', 10000, 'MaxIter', 10000, 'Display', 'none');

[fit, ~, flag] = fminsearch(@(x)Error(x, Freq_Subset, Ampl_Subset), x0, opts);

model.A = fit(1);

model.w = fit(2);

model.y0 = fit(3);

model.ok = flag;

model.fn = @(f)Model(fit,f);

end

function y = Model(x, f)

y1 = x(1) * exp(-f/x(2));

y2 = x(4) * exp(-(f-x(5))/x(6));

y = y1 + y2 + x(3);

end

function e = Error(x, f, A)

y = Model(x, f);

e = sum( (y - A).^2 );

end

Matlab function used to fit a Lorentzian curve to residuals from the dual-exponential fit (A). The parameter aPeakStartOptions is the initial peak frequency is estimated from guides manually applied to the waterfall plot (see text). Multiple options may be supplied; the one producing the best fit is chosen if more than one option is supplied.

function [model, AFit] = Fit_Lorentz(f, A, aPeakStartOptions)

for iStart = 1:length(aPeakStartOptions)

PeakStart = aPeakStartOptions(iStart);

% Fit to the supplied data

%sel = 0.5 <= f;

sel = 1 <= f;

Freq_Subset = f(sel);

Ampl_Subset = A(sel);

Height = max(Ampl_Subset) - min(Ampl_Subset);

x0 = [PeakStart, 2, Height/1e2, min(Ampl_Subset)];

opts = optimset('MaxFunEvals', 100000, 'MaxIter', 100000, 'Display', 'none');

[fit, ~, flag] = fminsearch(@(x)Error(x, Freq_Subset, Ampl_Subset), x0, opts);

result.fit(iStart, :) = fit;

result.flag(iStart) = flag;

result.error(iStart) = Error(fit, Freq_Subset, Ampl_Subset);

end

good = result.flag > 0;

if sum(good) > 0

idx = 1:length(aPeakStartOptions);

idxGood = idx(good);

[~, iMin] = min(result.error(good));

iMin = idxGood(iMin);

fit = result.fit(iMin,:);

flag = iMin;

else

fit = x0 * nan;

flag = 0;

end

model.x0 = fit(1);

model.w = fit(2);

model.A = fit(3);

model.y0 = fit(4);

model.ok = flag;

model.fn = @(f)Model(fit,f);

AFit = Model(fit, f);

end

function y = Model(x, f)

y1 = Lorentz(f, min(20, x(1)), abs(x(2)), abs(x(3))*1e2, 0);

y = y1 + x(4);

end

function e = Error(x, f, A)

y = Model(x, f);

e = sum( (y - A).^2 );

% Don’t let clila frequency outside of expected bounds.

freq.max = 20;

freq.min = 1.5;

e = e * penalize_out_of_range(x(1), freq);

% Don't let cilia frequency bump get too narrow or wide.

width.max = 20;

width.min = 1.5;

e = e * penalize_out_of_range(x(2), width);

ampl.max = 1000;

ampl.min = 0;

e = e * penalize_out_of_range(x(3), ampl)^2;

end

function scaler = penalize_out_of_range(v, range)

v = abs(v);

if v < range.min

scaler = (1 + abs(v - range.min));

elseif v > range.max

scaler = 1 + v - range.max;

else

scaler = 1;

end

end
